# Porous 8YSZ Ceramics Prepared with Alkali Halide Sacrificial Additives

**DOI:** 10.3390/ma16093509

**Published:** 2023-05-03

**Authors:** Julio Cesar Camilo Albornoz Diaz, Eliana Navarro dos Santos Muccillo, Reginaldo Muccillo

**Affiliations:** Center of Science and Technology of Materials—CCTM, Energy and Nuclear Research Institute—IPEN, Sao Paulo 05508-170, SP, Brazil; jccadun@gmail.com (J.C.C.A.D.); enavarro@usp.br (E.N.d.S.M.)

**Keywords:** yttria-stabilized zirconia, alkali halide pore formers, thermal analysis, impedance spectroscopy

## Abstract

8 mol% Y_2_O_3_-stabilized ZrO_2_ (8YSZ) ceramics were prepared with KCl and LiF additions to obtain porous specimens with high skeletal density. Thermogravimetric and differential thermal analyses (TG/DTA) were carried out on 8YSZ and on 8YSZ mixed to 5 wt.% KCl or 5 wt.% LiF as sacrificial pore formers that were thermally removed during sintering. The melting and evaporation of the alkali halides were evaluated by differential thermal analysis. Dilatometric analysis was also carried out following the same TG/DTA temperature profile with results suggesting rearrangement of the 8YSZ particles during LiF and KCl melting. The dilatometric data of 8YSZ green pellets mixed to KCl or LiF exhibited an initial expansion up to the melting of the alkali halide, followed by shrinkage due to sintering evolution with grain growth and pore elimination. The time that the alkali halide molten phase was kept during sintering was found to be an important parameter for obtaining 8YSZ-sintered specimens with specific pore content; bulk density and open porosity could then be tuned by controlling the time the alkali halide remained liquid during sintering. Scanning electron microscopy images of the pellet fracture surfaces showed pores that contributed to increasing the electrical resistivity as evaluated by impedance spectroscopy analysis.

## 1. Introduction

Sintering is one of the main thermal steps that has long been applied in the ceramics industry. During the process, modification to the microstructure of the materials occurs continuously, the control of which is necessary for obtaining functional ceramics [1]. Conventional sintering processes usually require high temperatures and a long time, but new approaches, like the ones using low-melting-point additives, have been proposed to produce ceramic materials with a controlled microstructure at lower temperatures and a shorter time, thereby reducing costs and eventually an improved final product [2].

Liquid-phase sintering is characterized by using a liquid phase to provide the interparticle bond of a solid particulate matrix with particle re-arrangement due to capillary forces. When both the liquid and solid phases interact, diffusion mechanisms take place that improve the final densification [3]. If the average size of the melt-forming particles is larger than the interparticle voids, pore formation can occur due to the persistent liquid phase and possible accumulation of gaseous species in the pores (even in closed pores swelling is expected), which promotes coarsening of the pores and inhibiting densification [3,4,5,6].

By controlling the processing of porous ceramics, a wide range of applications has been found, for example, as inorganic membranes [7,8] and in solid-oxide fuel cells [9,10].

Zirconia (ZrO_2_) is a paradigmatic ceramic material that finds application in several sectors of the economy. Even though zirconia ceramics have been studied thoroughly in recent decades such as at dedicated topic conferences [11], research is still being carried out to look for further applications. Zirconia may be found in three main crystallographic phases from room temperature to its melting point at approximately 2700 °C: monoclinic at room temperature, tetragonal from 900 to 1200 °C and cubic from 2370 °C to the melting point. The cubic phase may be stabilized at room temperature by constituting solid solution with aliovalent cations like Ca^2+^, Mg^2+^, Y^3+^ and RE^3+^ (Rare Earths) [10]. Zirconia stabilized with 8 mol% yttria (8YSZ) is a well-used ceramic material that has oxygen ion conductivity appropriate for use as a solid-electrolyte oxygen sensor in vehicles to improve engine efficiency and fuel economy [12,13,14,15,16], in high-temperature solid-oxide fuel cells for alternative electrical energy production [17,18,19,20] and in thermal barrier coatings [21,22,23,24].

Porous solid electrolytes (like 8YSZ) are particularly interesting because their physical properties have been widely studied [10] and find many applications [9]. Porous monoclinic zirconia has been proposed for use in separation processes for microfiltration with a pressure gradient [25]. The interdependence among sintering conditions, electrical properties, and porosity in liquid-phase, flash-sintered 8YSZ (with LiF as a sintering additive) has already been shown [26]; however, the sintering process in that case was faster due to Joule heating promoted by an electric current through the sample in an electric field. Porous 8YSZ is also used in in microporous membranes for gas nanofiltration [7] and carbon dioxide capture [27] and in thermal barrier coatings [28].

In this work, data from thermal analyses (thermogravimetry, differential thermal analysis and dilatometry) were collected in 8YSZ mixed to the alkali halides KCl or LiF (chosen due to their lower melting points, 770 and 845 °C, respectively, compared with that of ZrO_2_) to evaluate the thermal removal of the alkali halides as sacrificial pore formers. Impedance spectroscopy analysis was carried out after removal to determine the electrical resistivity of the porous 8YSZ specimens. Scanning electron microscopy images were collected for analysis of pore content. Even though porous ceramics can be obtained by different techniques (e.g., sacrificial polymers [29], coal-gang residual industrial waste [30]), the main objective here was to show that alkali halides could be used to obtain 8YSZ with controlled porosity using conventional sintering procedures: heating temperature and level-dwelling time-cooling steps.

## 2. Materials and Methods

### 2.1. Materials

ZrO_2_: 8 mol% Y_2_O_3_ ceramic powders (6.9 m^2^ g^−1^ specific surface area, 0.5–0.7 μm average particle size, 8YSZ, tape cast-grade powder, fuel cell materials, USA [31]) were dry mixed with 5 wt.% KCl (99.5%, Merck, São Paulo, Brazil) or with 5 wt.% LiF (99.5%, Oregon Labware, São Paulo, Brazil) pore formers, uniaxially pressed (50 MPa) into disk-shaped pellets having a 5 mm diameter and 3 mm thickness, and isostatically pressed (140 MPa, National Forge Co., Irvine, PA, USA). For collecting the FEG-SEM images and impedance spectroscopy data, the pellets were sintered in a furnace (Lindberg-BlueM, Watertown, NY, USA) with the following temperature profile: 1100 °C/1 h to remove the KCl or LiF, followed by 1400 °C/2 h with 5 °C min^−1^ heating and cooling rates, for densification. The density of the sintered samples was evaluated by applying the Archimedes principle, using distilled water as medium and an AG245 analytical balance (Mettler-Toledo, LLC, Columbus, OH, USA).

### 2.2. Thermal Analysis

Simultaneous thermogravimetric and differential thermal analyses (TG-DTA) were performed in the 8YSZ powder and in mixtures of 8YSZ with LiF or KCl, from room temperature to 1400 °C at 10 °C min^−1^, under flowing synthetic air at 10 L min^−1^ in a STA 409E (Netzsch, Selb, Germany) thermal analysis equipment; α-alumina was used as reference. For dilatometric analyses, data were collected by a green pellet inserted into the vertical dilatometer Unitherm 1161 (Anter, Pittsburgh, PA, USA), during heating to and cooling from 1400 °C at 10 °C min^−1^; The temperature was monitored by a type S thermocouple with its sensing tip positioned close to the sample.

### 2.3. X-ray Diffraction

X-ray diffraction data were collected in a Bruker-AXS D8-Advance diffractometer (Karlsruhe, Germany) with a θ–2θ Bragg–Brentano configuration with a scintillation detector, 40 kV–40 mA Cu-k_α_ radiation, in the 20–80° 2θ range, 0.05° step size at 5 s per step. The specimens were powders obtained by grinding the sintered pellets in an agate mortar. PDF files 30-1468 (8YSZ), 78-1217 (LiF) and 72-1540 (KCl) were used to analyze the diffration patterns.

### 2.4. Scanning Electron Microscopy

For observation in the scanning electron microscope, the sintered pellets were successively polished with 400-, 600- and 1000-grade SiC powders, followed by 15, 3 and 1 μm diamond pastes (Buehler, Lake Bluff, IL, USA), with further ultrasonic cleaning with isopropanol. The polished specimens were thermally etched at 1300 °C/15 min, inserted to and removed from the furnace with a 2 °C s^−1^ cooling rate using a platinum grid and leads.

The polished and thermally etched surfaces and fracture surfaces of samples were observed in a Inspect F50 FEG Scanning Electron Microscope (FEI, Brno, Czech Republic). The porosity level of the ceramic specimens was evaluated in two ways: (i) comparing the density determined by the Archimedes method with theoretical density [10]; (ii) signaling the pores in amplified scanning electron microscopy images and using the ImageJ software [32] to evaluate pore sizes and pore-size distribution. Energy Dispersive X-ray Spectrometer (EDAX Octane Elect Plus, Ametek, Berwyn, IL, USA) was used for the elemental searching of the sample surfaces for residual alkali halides after heat treatment to remove KCl and LiF in 8YSZ + 5 wt.% KCl and 8YSZ + 5 wt.% LiF, respectively.

### 2.5. Impedance Spectroscopy

In the [−Z″(ω) × Z′(ω)] diagrams, Z′ and Z″ are the real and imaginary components of impedance, respectively, and f = ω/2π is the frequency of the input signal. These were obtained by electrochemical impedance spectroscopy using a Hewlett Packard 4192A impedance analyzer (Yokogawa-Hewlett Packard, Tokyo, Japan) connected to a desktop workstation (series 360 HP Controller Yokogawa-Hewlett Packard, Tokyo, Japan) with a 200 mV input signal at 20 points per decade in the 10 Hz–10 MHz range. For the measurements, three samples were spring-loaded between platinum disks inside Inconel 600, alumina, and platinum terminal leads in a custom-made chamber connected to an impedance analyzer by 1 m coaxial cables. Silver paste was deposited on the parallel surfaces of the ceramic pellets and cured at 400 °C/15 min. Special software was used to collect and plot the [−Z″(ω) × Z′(ω)] data [33]. Deconvolution analysis of the diagrams was done with the same software, allowing for evaluating resistance and capacitance of the diagram semicircles.

## 3. Results

### 3.1. Density and Porosity Evaluation

The geometrical green density of the 8YSZ cold-pressed powders was approximately 45% of the theoretical density, (TD) [10]. The Archimedes density of the 8YSZ sintered pellets was 5.85 g cm^−3^ (98.2% TD). The 8YSZ sintered with KCl addition and 8YSZ sintered with LiF addition had approximately 78.9 and 56.2% TD, respectively, with corresponding 21.1 and 43.8% apparent open porosity.

### 3.2. Thermal Analyses

**Figure 1:**
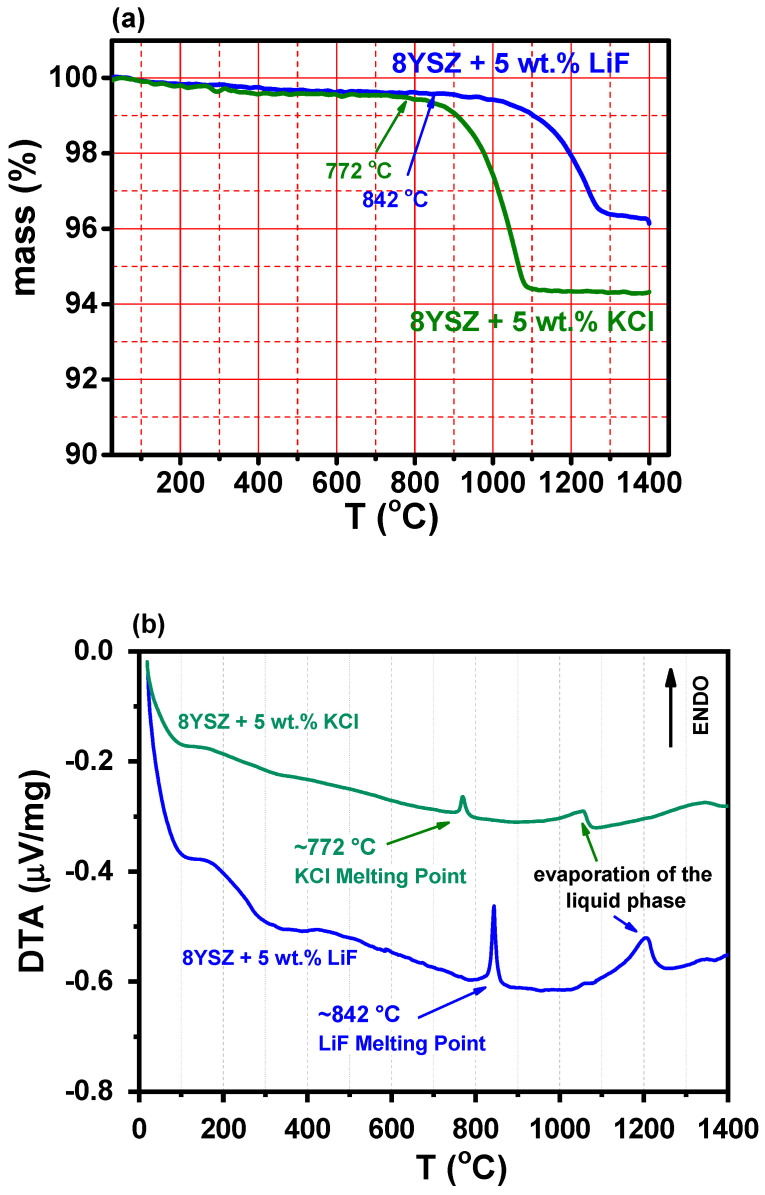
(**a**) Thermogravimetric and (**b**) differential thermal analysis curves of mixtures of 8YSZ + 5 wt.% KCl and 8YSZ + 5 wt.% LiF powders; heating rate: 10 °C min^−1^.

The thermogravimetric and differential thermal analysis curves during heating 8YSZ mixed to KCl or LiF at 10 °C min^−1^ to 1400 °C are presented in Figure 1a,b, respectively. The mass reduction after the specimen started melting was approximately 4 and 5% for LiF and KCl, respectively, meaning that a small amount of LiF might have remained in the sample. For both 8YSZ with KCl and with LiF, the differential thermal analysis showed endothermic peaks corresponding to the melting processes (approximately 772 and 842 °C for KCl and LiF, respectively, indicated by arrows), and wider high-temperature endothermic peaks associated with the evaporation of the liquid phase, approximately 1050 and 1200 °C for LiF, respectively, indicated by arrows), which was in agreement with the thermogravimetric data.

**Figure 2:**
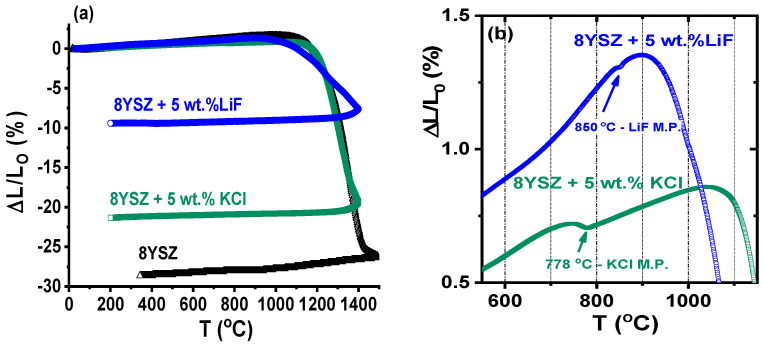
(**a**) Dilatometric curves of 8YSZ, 8YSZ + 5 wt.% KCl and 8YSZ + 5 wt.% LiF pressed pellets; (**b**) expanded view before sample shrinkage, pointing to the melting points of LiF and KCl; heating and cooling rates: 10 °C min^−1^.

Figure 2a shows dilatometric curves of 8YSZ, 8YSZ + 5 wt.% KCl and 8YSZ + 5 wt.% LiF cold-pressed pellets. The final attained shrinkage levels were 28.7, 21.3 and 9.4% for 8YSZ, 8YSZ + 5 wt.% KCl and 8YSZ + 5 wt.% LiF, respectively, indicating that the sintering additives inhibited shrinkage upon heating, probably by impeding pore removal and inhibiting grain growth. The dilatometry behavior depended on LiF and KCl melting due to the rearrangement of the 8YSZ solid particles—melting point higher than 2700 °C [10])—promoted by volume reduction during melting. Nearly negligible thermal expansions due to melting of KCl at 778 °C and of LiF at 850 °C were detected (Figure 2b), which agreed with the differential thermal analysis results in Figure 1. Moreover, shrinkage starts after melting and the partial or total thermal removal of the sintering additives allowed at least partial pore removal. If there is no densification because of the temperature, the additive becomes liquid until its removal, suggesting that the solubility of 8YSZ at both KCl and LiF liquid phases is negligible.

The differential thermal analysis data showed that KCl remained liquid from its melting point up to approximately 1050 °C for 8YSZ + 5 wt.% KCl. For 8YSZ + 5 wt.% LiF, this behavior occurred up to 1200 °C, the temperature reached when the sintering of 8YSZ would have already started. In both cases, the capillary forces exerted at the ceramic particles by the molten sintering additives allowed for 8YSZ particle rearrangement and pellet sintering; however, one should consider that the liquid phase may have remained partially undissolved in the bulk of the specimens, while its vapor pressure and its fluidity increased, thus promoting the formation of pores and consequently the build-up of a skeletal 8YSZ structure. At the end of the sintering process, the remaining time when the liquid phase and the 8YSZ sintering processes overlapped (longer in the sample with LiF) controlled the level of porosity of the sintered sample after the removal of the sintering additives by thermal action, as suggested by the dilatometry results (Cf. Figure 2a). From this point of view, the final bulk density and open porosity of the samples could apparently have been controlled by adjusting the dwelling time of the liquid phase during sintering, thus allowing for the sintering of ceramic ionic conductors with the approximate desired porosity (e.g., dense and porous specimens for solid electrolytes and anodes in solid-oxide fuel cells [20]).

### 3.3. Microstructural Analyses

**Figure 3:**
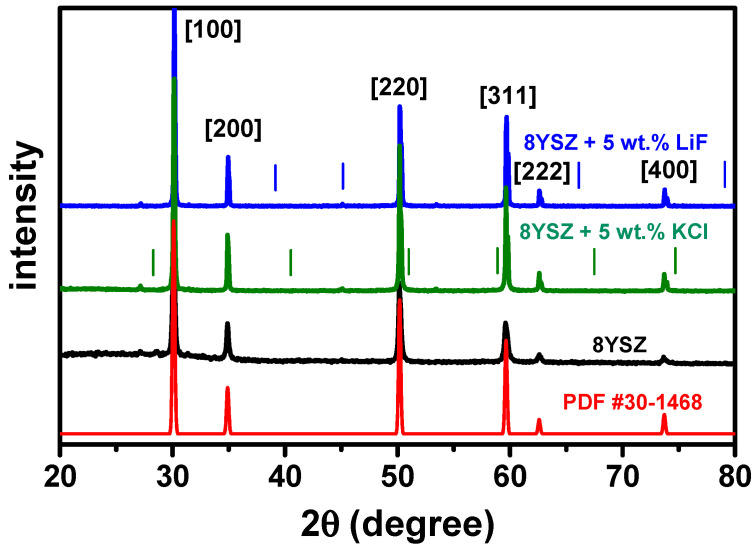
X-ray diffraction patterns of 8YSZ, 8YSZ + 5 wt.% KCl and 8YSZ + 5 wt.% LiF powders obtained by crushing pellets sintered at 1400 °C/2 h. Miller indices and PDF #30-1468 data are also shown, corresponding to cubic 8YSZ; vertical bars show positions of LiF (blue) and KCl (green) XRD reflections.

X-ray diffraction patterns of powders obtained by grinding the sintered pellets are shown in Figure 3. The data are in agreement with PDF #30-1468, corresponding to cubic 8YSZ. According to the X-ray diffractograms, KCl and LiF with their peak reflections signaled in Figure 3, were apparently removed completely from the samples, leaving only the 8YSZ ceramic matrix. Additional energy dispersive X-ray experiments were performed to confirm this result.

**Figure 4:**
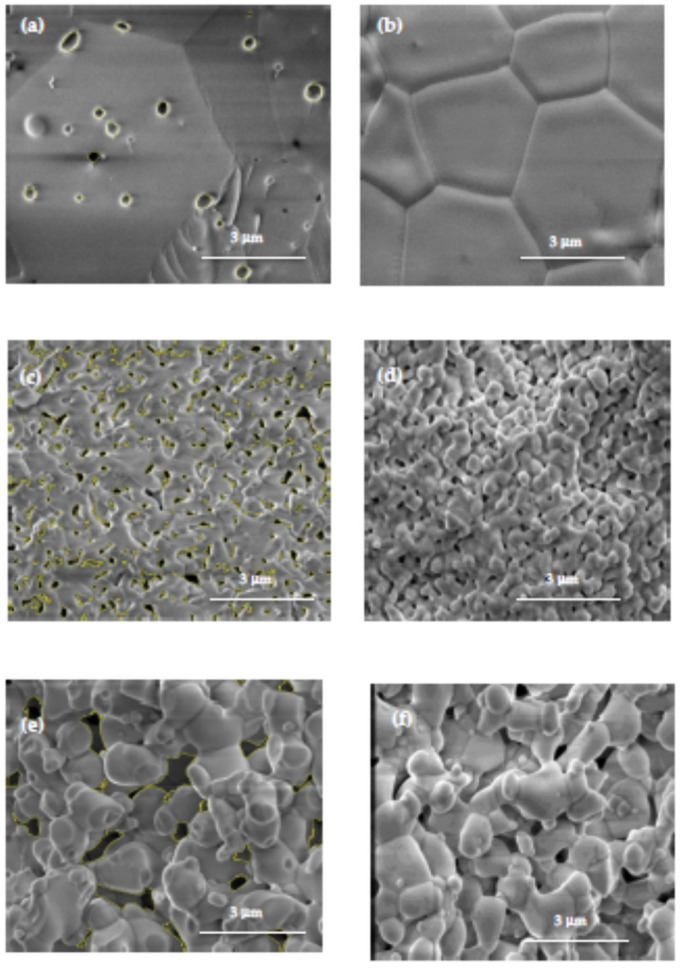
Scanning electron microscopy images of fractured (left) and polished + thermally etched (right) surfaces of 8YSZ (**a**,**b**), 8YSZ + 5 wt.% KCl (**c**,**d**) and 8YSZ + 5 wt.% LiF (**e**,**f**) sintered pellets. The black spots in the SEM images are the selected pores for constructing Figure 5.

Figure 4 shows FEG–SEM images of the fractured and polished surfaces of 8YSZ, 8YSZ + 5 wt.% KCl and 8YSZ + 5 wt.% LiF sintered pellets. The 8YSZ pellet surfaces presented high density, some inter- and intragranular porosity in the bulk (Figure 4a) but none at the external surface (Figure 4b), and a >3 μm average grain size with polygonal shape. The images of the 8YSZ pellet, sintered at 1400 °C to remove the KCl (Figure 4c,d) shows neck formation among grains in the submicron range and rounded grains, typical of liquid phase sintered ceramics. The 8YSZ sample, which had LiF as the sintering additive (Figure 4e,f), showed a different behavior: wide distribution of welded grains, predominant submicron grain sizes, pores and dense skeletal framework with neck formation among grains.

According to these images, the difference in the microstructure of 8YSZ samples sintered with the additives was probably related to fouling, a reaction known to occur in the liquid phase with the zirconia–yttria particles. In the specimen with the LiF addition, the alkali halide remained partially in the sample at relatively higher temperatures than for KCl, and the grains achieved a higher than average size (~1 μm).

**Figure 5:**
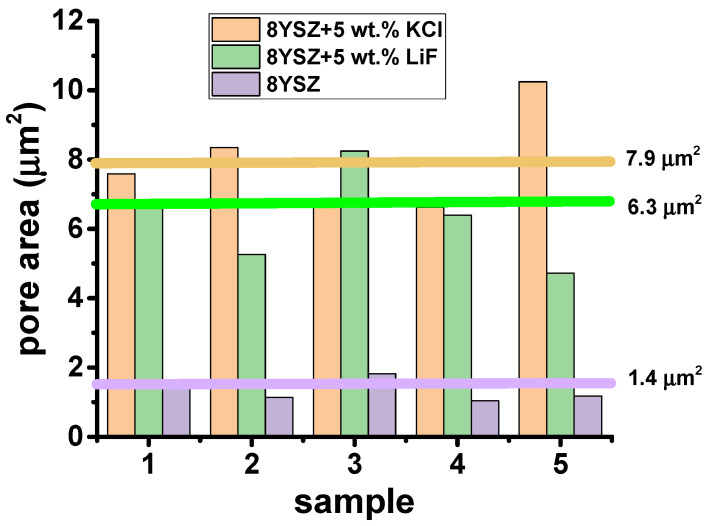
Pore areas of 8YSZ-, 8YSZ + 5 wt.% LiF- and 8YSZ + 5 wt.% KCl-sintered samples evaluated using ImageJ software [32] in SEM micrographs measured at different locations on the sample surfaces.

**Figure 6:**
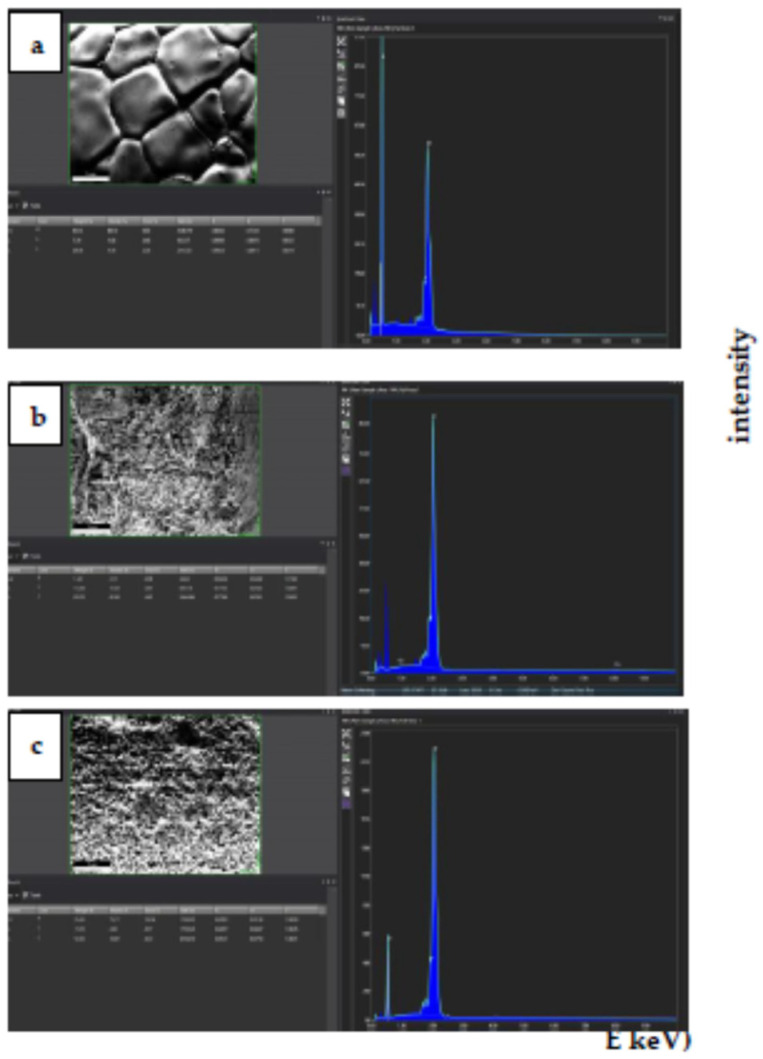
EDX images of surfaces of (**a**) 8YSZ-, (**b**) 8YSZ + 5 wt.% KCl- and (**c**) 8YSZ + 5 wt.% LiF-sintered pellets.

**Figure 7:**
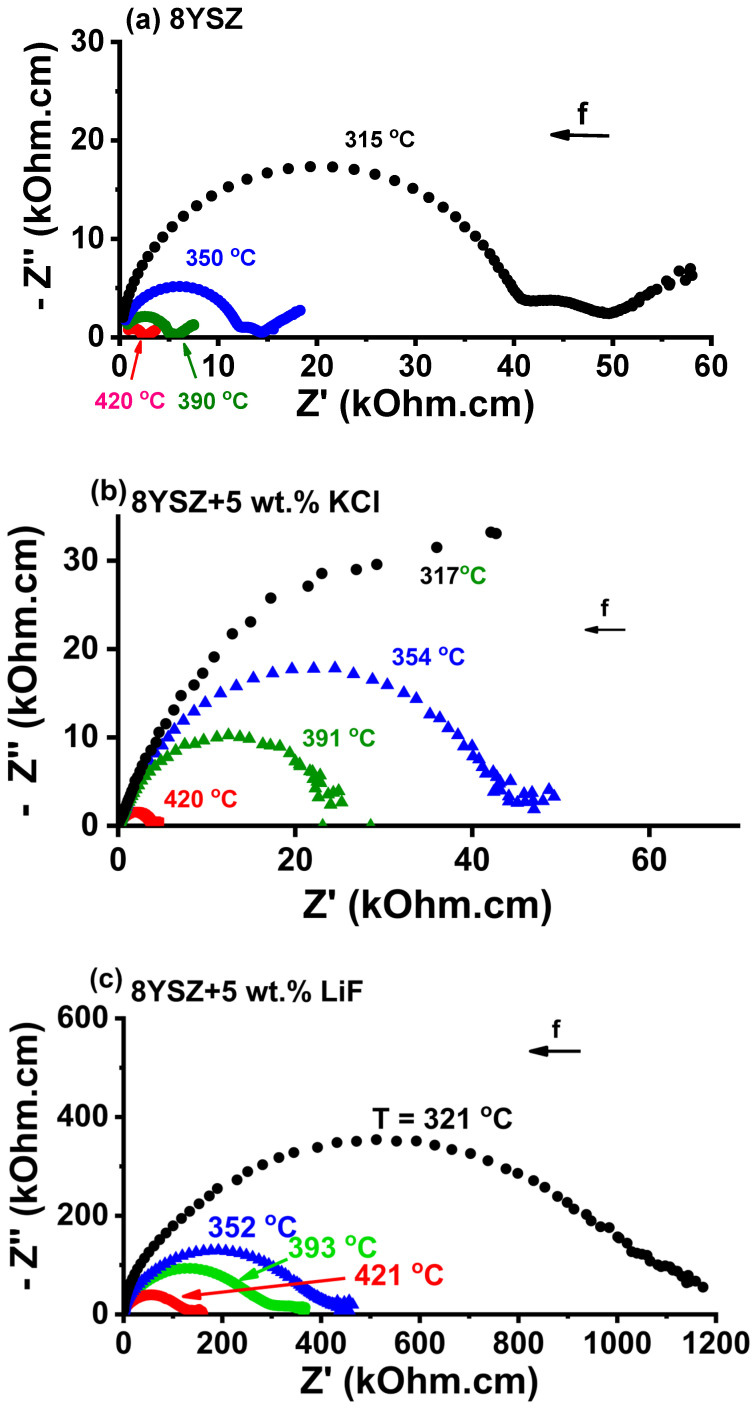
Impedance spectroscopy diagrams of (**a**) 8YSZ, (**b**) 8YSZ + 5 wt.% KCl and (**c**) 8YSZ + 5 wt.% LiF pellets sintered at 1400 °C/4 h.

Additional scanning electron microscopy images were collected at different regions of polished and thermally etched surfaces of 8YSZ, 8YSZ + 5 wt.% KCl and 8YSZ + 5 wt.% LiF-sintered specimens. ImageJ software [32] was applied to evaluate the areas of selected pores in 5 images collected at different regions for each sample. The distribution and the average size of the pore areas are plotted in Figure 5. These values are 7.9, 6.3 and 1.4 μm^2^ for 8YSZ + 5 wt.% KCl, 8YSZ + 5 wt.% LiF and 8YSZ, respectively. These values are consistent with the total electrical resistance of the samples (Cf. Figure 6 and Figure 7): the larger the average pore area the higher the total electrical resistance.

The search for the residues of KCl and LiF in 8YSZ + 5 wt.% KCl- and 8YSZ + 5 wt.% LiF-sintered pellets was conducted by EDX analysis. The results are shown in Figure 6.

The three spectra, obtained from collecting data over a long time (10 min), are quite similar without any trace of potassium or chlorine (Figure 6b) or fluorine (Figure 6c). Oxygen (0.510 keV, k_α1_), yttrium (1.920 keV, L_α1_), and zirconium (2070 keV, L_α1_) were the detected elements. 

### 3.4. Impedance Spectroscopy

**Figure 8:**
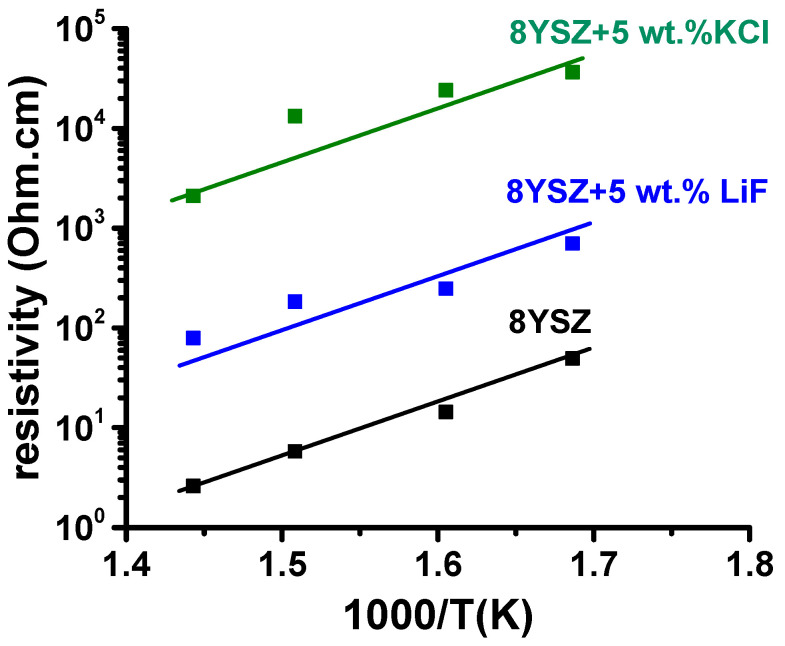
Arrhenius plots of the total electrical resistivity of 8YSZ, 8YSZ + 5 wt.% LiF and 8YSZ + 5 wt.% KCl after sintering at 1400 °C/2 h.

Figure 7 shows impedance spectroscopy diagrams of 8YSZ, 8YSZ + 5 wt.% KCl and 8YSZ + 5 wt.% LiF pellets. The values of the total electrical resistance R were collected at the intersection of the high frequency semicircle at the low frequency side of the impedance diagrams. Considering the geometric factor S/t, where S is the electrode area and t is the pellet thickness, the electrical resistivity values (ρ = R.S/t) at 390 °C were evaluated: 24.0, 24.2, and 186.5 kΩ.cm for 8YSZ, 8YSZ + 5 wt.% KCl and 8YSZ + 5 wt.% LiF pellets, respectively. The higher electrical resistivity of the specimens sintered with additives, comparing to 8YSZ reflected the contribution of pores to the increase in resistivity [34]. Porosity is known to contribute to the increase in the electrical resistivity of stabilized zirconia as a consequence of the higher path to be pursued by the oxygen ions to circumvent the pore surface [34]. Arrhenius plots of the collected values are shown in Figure 8. An activation energy of 1.1 eV was evaluated for the electrical conduction of oxygen ions, which was in agreement with published values (~1 ([2] p. 9), 1.03 [35] and 0.98–1.11 eV [36]). The same activation energy meant that the charge carrier (O^2−^) was the same; higher resistivity meant that the O^2−^ percolation path was larger due to the existence of pores to be circumvented.

The overall results showed the possibility of using either potassium chloride or lithium fluoride as sacrificial pore formers to prepare, by conventional sintering, zirconia that was fully yttria stabilized to be used as matrix in, e.g., dual phase membranes for chemical species capture [28] or thermal barrier coatings [29].

## 4. Conclusions

Porous 8YSZ ceramics were obtained using KCl and LiF as pore-forming sacrificial sintering additives. Thermogravimetric data indicated the nearly complete thermal removal of KCl and LiF. Simultaneous differential thermal analysis showed two endothermic peaks associated with the melting process of the sintering additives and their evaporation. Thermal dilatometry measurements exhibited a final attained shrinkage for 8YSZ-, 8YSZ + 5 wt.% KCl- and 8YSZ + 5 wt.% LiF-pressed pellets, respectively, of 28.7, 21.3 and 9.4%, indicating that the sintering additives inhibited densification. Moreover, during the thermal expansion before sintering, the melting of LiF and KCl was evident. The thermal experiments suggested that the final bulk density and the open porosity could be managed by controlling the time the liquid phase remained during sintering. Scanning electron microscopy images of the surfaces of the sintered samples showed that the 8YSZ specimens sintered with KCl and LiF had average grain sizes much smaller than that of the 8YSZ specimen. Impedance spectroscopy analysis showed higher electrical resistivity of the 8YSZ samples that had been sintered with the alkali halide additions, which was in agreement with the dilatometry and SEM results.

## Data Availability

Not applicable.
